# The Context-Variable Self and Autonomy: Exploring Surveillance Experience, (Mis)recognition, and Action at Airport Security Checkpoints

**DOI:** 10.3389/fpsyg.2019.02258

**Published:** 2019-10-22

**Authors:** Meghan E. McNamara, Stephen D. Reicher

**Affiliations:** School of Psychology and Neuroscience, University of St Andrews, St Andrews, United Kingdom

**Keywords:** airports, autonomy, identity claims, frame of reference, recognition/misrecognition, selfhood, social identity, surveillance

## Abstract

This paper critiques and extends the notion of autonomy by examining how common autonomy definitions construct selfhood, with the support of an analysis of airport surveillance experiences. In psychology, autonomy is (1) often oriented around volition and action rather than the-self-that-acts and (2) the-self-that-acts is construed in singular terms. This neglects the multiple, context-variable self: while others may confirm our self-definitions (recognition), identity claims may also be rejected (misrecognition). The autonomy critique is sustained through an ethnographic analysis of airport security accounts (*N* = 156) in multiple nations with comparable security procedure (e.g., identification checks, luggage screening, questioning). Such procedures position people in multiple ways (e.g., as safe/dangerous, human/object, respectable/trash). Where respondents felt recognized, they experienced the security procedures positively, actively assisted in the screening process (engaged participation), and did not adapt their behaviors. Where respondents felt misrecognized, they experienced surveillance negatively, were alienated, and responded by either accommodating their behavior to avoid scrutiny, seeking to disrupt the process, or else withdrawing from screening sites. In misrecognition, the strategies that are open to the subject are incompatible with autonomy, if autonomy is defined solely in terms of volition. Accordingly, the concept of autonomy needs to be analyzed on two levels: in terms of the subject’s ability freely to determine their own sense of self, as well as the actor’s ability freely to enact selfhood.

## Introduction

Surveillance is a pervasive part of the contemporary world (Lyon, 2018). It is embedded in everyday life (Green and Zurawski, 2015), unless you go completely ‘off the grid’ (Joh, 2013). And yet, its significance to psychological research has long been underestimated (Ellis et al., 2016), despite the fact it has a long history (cf. Gilliom, 2001; Choudry, 2019). This is a critical deficit, given that surveillance has been characterized as ‘ubiquitous’ (Andrejevic, 2012), ‘intensifying’ (Ball and Webster, 2003; Jeffreys-Jones, 2017), and takes many forms. To name but a few, companies classify potential consumers (Pridmore and Zwick, 2011), employers monitor productivity (Ball, 2003), and government and non-government organizations intercept cellular phone communication (Pell and Soghoian, 2014). In all of these forms, and many others besides, surveillance observes and analyzes characteristics and action.

A good illustration of this can be seen in the novel, *The Circle* (Eggers, 2013), which illustrates a speculative, near-future society filtered through a surveillance culture lens (Lyon, 2018). There is but one possible self in *The Circle*, that of the enthusiastically open and participatory Circle citizen, and it must always be legible to the system (see also, Martin, 2010). So, Mae, a Circle employee, becomes fully transparent. She enthusiastically records and broadcasts her daily life. In the course of live streaming, however, she exposes one of her parents’ intimate moments to Internet viewers (see also, Ball, 2009).

While Mae can act autonomously within the *Circle*, her parents can’t. By the end of the story, she hadn’t heard from them in months. Despite this, she assumed “it would only be a matter of time (Eggers, 2013, p. 497).” The impression we are left with is, should contact be reestablished, it would not be willing on her parents’ part. And yet, Mae remains oblivious. She assumes she understands their selfhood and actions, but she does not.

This example demonstrates two things. First, trying to understand action with an inadequate model of selfhood is a risky proposition. Second, autonomy requires the ability to act from the self one chooses. As such, it also illustrates a fundamental assumption that exists within popular academic autonomy definitions: that there is but one self that acts.

Self-Determination Theory (Deci and Ryan, 1985) provides a good example. Here, “autonomy refers…to the feeling of volition that can accompany any act (Ryan and Deci, 2000b, p. 74).” The core arguments focus on motivated action, that is, whether you do something because you want to do it (autonomy), because of an external reward or punishment (heteronomy) (e.g., Ryan and Deci, 2000a), or because you decide the behavior is meaningful (identification) (e.g., Ryan and Deci, 2012).

Autonomy is therefore intimately bound up with selfhood. However, the nature of selfhood is underexplored in this literature. Here, autonomy definitions are structured around action, and assume selfhood. In order to understand autonomy, we must also understand the self that acts.

These are the issues explored in this paper, in the context of airport security screening:

(1) The role of selfhood in autonomy, by problematizing ‘self’ and ‘autonomy’;
(2) The experience of others perceiving self-definitions that are consonant (recognition) or dissonant (misrecognition) with our own;
(3) How people respond and behave after recognition/misrecognition;
(4) The analytical implications for autonomy under conditions of recognition/misrecognition.

As demonstrated in the analysis, airport surveillance observes people and then sorts them into categories. It defines them as ‘safe’ or ‘dangerous,’ as ‘one of us’ or as an ‘enemy to us.’ Both are concerned with recognition and may lead people to feel misrecognized. What does this mean to those under scrutiny, and what does it tell us about autonomy?

### The Relationship Between Selfhood, Context, and Action

As intimated, the prevailing psychological autonomy literature has under-theorized selfhood. For instance, Self-Determination theorists define the self as “the psychological manifestation and extension of the organizational properties common to all living things (Ryan, 1991, p. 214).” The self is described as the “set of coherently organized processes, structures, and energies that are the developmental outcome of organisimic integration (Ryan et al., 2015, p. 801).” This opens up, but does not address, a series of possibilities around selfhood. This paper examines just one set of issues, which will be central to the subsequent argument: whether selfhood is single or multiple, and the significance of context.

First, although the research that investigates action, like Self-Determination Theory, implies that multiple selves are possible (e.g., Ryan and Deci, 2012), it is not made clear how these selves are structured, or how they function, as a part of human experience (McConnell, 2011; McConnell et al., 2012). As a result, there is a lack of attention to contextual variability and the consequences of interacting with social norms and structures.

This inattention to the multiple self and social practice is not unique. Much of the psychological literature conceives of selfhood as singular and context-independent. A constant, ‘core’ self is assumed to remain unchanged between situations and across time, which is not the case (cf. Onorato and Turner, 2004). Even though the possibility of multiple selves is mentioned in passing, “the impression derived from the literature suggests that there is a single self (McConnell, 2011, p. 4).”

Second, the autonomy literature has not been explicit about how selfhood relates to context, and whether it varies as a consequence of context. Instead, context is addressed in the delimited sense of whether it is controlling or autonomy supportive (e.g., Ryan and Deci, 2012). However, contexts are structured by human perception, practices, and sense-making (Shweder, 1995). As a result, positionality (characteristics like identities and location) affects how we see the world (Sánchez, 2010). It follows that understanding how people experience and react to situations may well require understanding multiple contexts.^1^

This, in turn has implications for understanding action. Linking action to the self without explicitly relating selfhood to context inevitably compromises the ability to theorize autonomy. Conceptually integrating the context-variable self into how autonomy is defined presents a solution.

### The Variable Self and Its Implications for Action

The notion of the variable self has acquired prominence in psychology through the social identity tradition (Tajfel and Turner, 1979), and more recently through self categorization theory (Turner et al., 1987), which is distinctive precisely for the way in which it links a variable self-concept to the changing structure of the various contexts in which we live (Turner et al., 1994). Briefly, social identity theorists argue that the self is not a unitary construct, but rather a system in which we have many identities that vary as a result of context. While some may choose to identify with, say, a political party or a favorite sports team, not all categories are freely chosen, and not all claimed identities are recognized by others. This is crucially important, as misrecognition constrains action (Hopkins and Blackwood, 2011; see also, White, 2002).

But this is not all. A variable self reading of the social world also reveals a critical blind spot in psychology research design, which has implications for how the previous autonomy literature should be analytically understood. While the ways in which we are constrained by others is acknowledged in principle in psychology, it is often ignored in practice in a domain where experiments are the dominant research method.

As Howarth (2002) argued in reference to social identity and self-categorization research in particular, experiments construct a world where “the picture is of each and every individual constructing an identity on [their] own, choosing where to position [themself], cut off from the influence of and pressure from others (p. 157).” This critique is applicable to most quantitative research on identity in psychology. In most circumstances, researchers analyze identity in terms of how people self-identify. Identity is treated as a choice, rather than as a claim. Generally (though not always: see Hopkins et al., 2015), there is no attention paid to whether these claims are accepted by others. Even when participants are allocated to conditions randomly, these biases still exist when investigators talk about identity in relationship to variables that are determined by how participants filled out their demographics questionnaires.

Other researchers, however, have shown clearly that it is better to regard identity as a claim rather than a choice (Joyce et al., 2013; Joyce and Lynch, 2018; Moffitt et al., 2018). Whether the claim is accepted or not is far from assured. As a result, the match or mismatch between how I see myself (or wish to be seen) and the ways others see me becomes crucial to whether action is constrained or autonomous. Let us therefore explore it further.

### Recognition and Misrecognition Are Centrally Important to Understanding Action

The rift between imposed and accepted self-definitions can be understood through the concepts recognition and misrecognition. To be clear, the term ‘self-definition’ describes both imposed (hetero self-definition) and self-generated (auto self-definition) categories that are used to label parts of the self-complex. Self-definitions are phenomenological and can be observed directly (e.g., through self-report). Therefore, they should not be confused with self concepts, which are “hypothetical cognitive structure[s] which cannot be observed directly (Turner et al., 1987),” or self aspects, which, as a term, has been positioned to include both hypothetical as well as phenomenological categories (see, for instance, McConnell, 2011).

Recognition occurs when an auto self-definition is acknowledged, reinforced, or accepted by others. Here, there is consonance between how I see myself and how you see me. Misrecognition occurs when these two diverge: the auto self-definition is either denied or else given a different value or meaning by others. It is instead a hetero self-definition, rather than an auto self-definition.

Recognition and misrecognition have important consequences. This can be seen clearly in work conducted by Blackwood and colleagues, who explored how Muslims see themselves as Scottish but are treated as ‘other’ when passing through airports. These participants experienced their Muslim identity as conferring respectability, but others treated it as a sign of danger (Blackwood et al., 2013, 2015).

So, misrecognition can be a painful experience. But it’s not simply unpleasant. It may impact negatively on relations between the misrecognized and those who commit misrecognition, because it can have profound effects on the ways in which people feel able to communicate and act (Hopkins and Blackwood, 2011). If people are unable to act on their own sense of self, they can either act upon an imposed selfhood or else to try and reassert their original selfhood.

In either case, simply acting on their auto self-definition is no longer an option. So, recognition is arguably essential to autonomy (see also, Anderson and Honneth, 2005; Renger et al., 2017).

Finally, as explained, the self is multiple and contextually variable. As such, autonomy becomes not just a matter of acting upon the self, but also a matter of choosing the self upon which one acts, and having that choice recognized. The variable, (mis)recognized self thus presents clear implications for autonomy, in terms of both experience and perception.

### Exploring the (Mis)recognized Self and Action in the Context of Surveillance

In order to examine these issues, the domain of surveillance is ideal. Perhaps the clearest example of this in contemporary society is at the airport. Here, surveillance is both ubiquitous and explicit. We are watched, scanned, processed, sorted as we pass through passport control, customs, and airport security checkpoints. These classify us into different categories at different points (see also, Lyon, 2003). We are nationals or we are foreigners as we present our passports. We are honest or dishonest as customs personnel scrutinize us. We are safe or we are dangerous as camera operators look for suspicious signs. At all these points, and many others, the way we are defined by the surveillance process, and those who enact it, may either match or mismatch our own self-definitions.

Pat-downs, or frisking searches, are a good example. The Transportation Security Administration (TSA), the organization responsible for conducting airport screening in the United States, said in a recent training manual, “all standard pat-down searches must be conducted by a [Transportation Security Officer] of the same gender. An individual’s gender is what *he* or *she* purports *himself* or *herself* to be [emphasis added] (TSA, 2018, p. 13).” Here, the TSA (2018) offered its trainees two gender categories in which to understand how to conduct what they termed “same gender screening (p. 13).” But what if your gender identity doesn’t fit into one of those categories?

Pat-downs are just one of many possible situations where misrecognition may occur, and gender is just one of many possible identity dimensions along which misrecognition is possible. Previous recognition/misrecognition research looked at cultural identity (Ingram, 2009; Sametband and Strong, 2018), military identity (Molendijk, 2018), national identity content (Hopkins et al., 2015), and the experiences of minoritized groups in multiple contexts (Taylor, 2007; Hopkins, 2011; Hopkins and Blackwood, 2011; Habibis, 2013; Hopkins and Greenwood, 2013; Horton, 2014; Wills, 2014; Hopkins et al., 2017), including airports (Blackwood et al., 2013, 2015).

The present study was not limited to any specific category or any specific aspect of the airport experience. The sample was oriented around ‘air travelers’ generally (contrast with Blackwood et al., 2013, 2015), so as not to assume what categories might be relevant in advance. This left open the possibility of finding something unexpected, unbounded by a preconceived framework.

More specifically, the analysis addresses three questions:

(1) What are the forms of recognition and misrecognition experienced at airports?
(2) How do people experience these forms of recognition and misrecognition?
(3) How do people respond to different forms of recognition and misrecognition?

The analysis of these three questions leads to a fourth, which the findings suggest, and which is drawn together explicitly in the discussion. This relates to the central concern of this paper. That is, what are the implications of recognition/misrecognition for understanding autonomy?

## Materials and Methods

### Participants

Fifty-eight participants (*N* = 39F; *N* = 19M) provided airport security checkpoint experiences. This sample was a mix of students (*N* = 34) and non-students (*N* = 24) who reported multiple nationalities and places of residence. They ranged in age from 19 to 66 (median 25), and 68% (*N* = 40) had completed a Bachelor’s degree.

Those who were employed reported diverse occupations; two participants were retired, and one was an unemployed recent graduate.

Nearly two thirds (*N* = 36) self-categorized as White/Caucasian (*N* = 36);^2^ others reported Asian (*N* = 1), Asian Indian (*N* = 1 from North India), Caucasian and American Indian (*N* = 1), Chinese (*N* = 1), German and Cuban (*N* = 1), Latin American (*N* = 1), Scottish and Indian (*N* = 1), Taiwanese (*N* = 1), Vietnamese (*N* = 1) in this category. One person said ‘British,’ one provided ‘Scottish,’ and three did not provide this information.

The first author did not ask participants (*N* = 8) to provide race/ethnicity when she collected data in Latvia. A local research associate explained this question would not be a pertinent descriptor for participants who lived there. First language was requested as a proxy, as this indicates local majority (Latvian first language) or minority (Russian first language) group membership (Russian, *N* = 8). More detail on all of these categories can be viewed in the Supplementary Materials.

### Data

This study’s unit of analysis was airport security experience accounts (*N* = 156: airport security checkpoint, *N* = 153; airport security avoidance, *N* = 2; an encounter characterized as “no security check” when airport staff only checked passport and boarding pass; *N* = 1). Of the two people who reported airport security avoidance, one provided six accounts that occurred before the screening process introduced body scanners. Most (57%; *N* = 33) participants reported two accounts (Median *N* = 2; Min *N* = 1; Max *N* = 15). Some participants, however, talked about experiences in summary. Therefore, it would be accurate to say that this dataset refers to *N* > 156 airport security experiences.

Most (83%) of these surveillance experiences took place in Europe (*N* = 71) and the United States (*N* = 58). Most sites checked identification and bodies, processed luggage, and questioned travelers (*N* = 1 airport conducted no questioning, body or luggage screening for an internal, scenic flight). Reported airport security procedures were comparable across these sites in terms of general categories (e.g., identification, body, and luggage screening), though there were differences in terms of specific procedure.

As one participant observed, “there are inconsistencies in the script and choreography” between security checkpoints. For instance, participants reported that screening authorities emphasized different details between sites (e.g., either X-raying laptops in sleeves, or without any cover), but kept the broad strokes similar (e.g., those laptops were removed from carry-on bags so that both the computer and bag could be screened separately). More information about these data can be found in the Supplementary Materials, including the national locations where screening occurred.

### Sampling and Data Collection Procedure

The first author pulled these surveillance accounts from minimally structured interviews (*N* = 20) and structured diaries (*N* = 109)^3^ that asked travelers to report and reflect on their airport security experiences. Diversity sampling (in terms of participants and airport site) directed initial data collection, in order to explore security screening with limited preconceptions (e.g., who might have problems, or why those problems might exist). Theoretical sampling guided subsequent data collection, in order to explore initial themes, flesh out and saturate analytical categories, and challenge developing category fit.

The first author collected these data in person, via Skype, phone, text, email, and through a departmental office drop-box between 2010 and 2018. She collected these data in four stages, three targeted and one opportunistic (e.g., when someone spontaneously shared a story that filled a gap in the dataset, the first author asked them to participate in the study). Ethics approval for the first round of data collection was provided by the Department of Sociology at Cambridge. The School of Psychology and Neuroscience at St Andrews granted ethics approval for subsequent data collection.

In both the diaries and interviews, the first author asked participants to narrate screening encounters at airports and then reflect on them. Where necessary, she asked follow-up questions in order to clarify those answers. She recorded and transcribed most interviews. In cases where recording was not possible, she transcribed and paraphrased quotes during or immediately after interviews, and then wrote detailed notes soon after the interview ended.

The first sample’s interviews (*N* = 12; one interview included two participants) included participants recruited after travel (*N* = 13 participants; *N* = 39 airport security experiences; Min = 1, Median = 2, Max = 6). Interviewees were found through word of mouth and opportunistic meetings. The first author explained her interest in surveillance with the term “travel security experiences,” and let participants talk about what they found important. Each interview was tailored to the participant’s specific context and communicative preferences. Some participants shared what they were interested in starting with in advance. Those who did not provide a topic received prompts (“e.g., please tell me about your travel security experiences”).

For the second sample, participants (*N* = 36) were recruited in advance of travel. This occurred through a call for participants issued to two British University email lists in December 2011, through conversation (December 2011; February 2012), and through participants sharing the study others (February–April 2012). At total of 79 people received diaries (response rate = 45.6%). One person, a military member, had participated in an interview in 2010, and was also asked to provide diaries during travel in 2012 due to theoretical sampling.

This procedure resulted in *N* = 103 diaries (Min = 2, Median = 2, Max = 13), and *N* = 5 follow-up interviews (one interview included two participants). Most of the latter included people whose diaries either needed clarification or included characteristics otherwise unique to the dataset. One participant was not comfortable with writing but still wished to participate, so the first author read her the diary questions, recorded her responses, and asked follow-up questions immediately, as this participant knew the interviewer and used shorthand like “you know how I am” that needed explicit clarification.

Participants were asked to fill out their diaries as soon as possible after travel. Diaries were used to balance the retrospective interviews; at the time of data collection, there was no way to know whether there would be analytically relevant differences in terms of how people reflected on their security experiences retrospectively or soon after travel. In addition, having multiple, mixed-method samples facilitated balance: using both diaries and interviews addressed the risk of over-sampling memorable outlier encounters, and facilitated a search for divergent cases.

In order to confirm the quality of the dataset, the first author also collected a third sample (*N* = 12 participants; *N* = 14 airport security experiences: Min = 1, Median = 1, Max = 2). This included retrospective diaries from students at a university in Latvia (*N* = 8) and retrospective interviews from an opportunistic sample *N* = 4. Two of these participants took part in the 2010 interviews: one was interviewed again because she socially shared a different type of experience, and the other was asked to reflect back on the following statement she reported in 2010, as it was germane to the developing analysis:

“When I feel impatient with all the procedures and waiting in lines I try and remind myself that they are just doing their jobs (Catherine, 56F, American).”

This framing was still entirely familiar to her in terms of how she usually experienced airport security (and when asked again during the paper write-up, she said, “yes, I still feel that way”). The 7-year-gap between collecting the initial account in 2010 and the follow-up questions in 2017 presented no challenge in terms of reflection because this framing was still relevant to her contemporary experience.

All of the data collection conducted outside of the first two waves was undertaken to challenge, and then ensure, thematic and theoretical saturation (Saunders et al., 2018). The first author stopped collecting airport security accounts when it was clear that experiences started repeating (thematic saturation). Since current dataset was also theoretically saturated with regard to the specific forms of recognition/misrecognition covered within this study, the dataset as a whole was deemed sufficiently large for the main analysis.

### Analytical Procedure

This analysis is centered on the experience and consequences of recognition and misrecognition during surveillance at airports. Like Pütz (2012), this study used understanding gained through ethnographic practice in order to analyze these data. However, unlike Pütz (2012), the first author targeted her observation to pre-planned travel (e.g., experiencing security as an analyst and traveler simultaneously on some occasions, and on others simply participating in the screening, with minimal targeted observation, and reflecting back later), rather than arranging for access to observe checkpoints purely as a researcher. Moreover, in contrast to Pütz (2012), this paper’s data are not limited to the authors’ own observation and reflection.

The airport security accounts (*N* = 156) were analyzed in two stages. The preliminary adaptive thematic analysis procedure (Table 1) was general and open. It was done in order to better understand airport surveillance experience as a whole, and then to discern concepts for future study. The main analysis was focused around one of those findings, recognition/misrecognition, which was explored with a procedure where airport security was treated as a text alongside the surveillance experiences (Table 2). More information on the analytical method, including explanation of communicative choices made during the analysis write-up, can be found in the Supplementary Materials (see also, Levitt et al., 2017, p. 5).

**TABLE 1:** Adaptive thematic analysis procedure.

**Step number**	**Analytical stage**	**Level of rigidity**	**Source**
1	Standardize units of coding	Fluid	Boyatzis, 1998
2	Familiarization	Fluid	Boyatzis, 1998; Braun and Clarke, 2006
3	Open coding Pattern recognition: “The ability to see”	Fluid	Braun and Clarke, 2006; Charmaz, 2006
4	Emic coding of experience type	Fluid	e.g., Maxwell, 2013; Harris, 1968
5	Connection mapping	Fluid	*Developed in situ*
6	Auditing of open and emic coding	Set	Influenced by Boyatzis, 1998; Elliot et al., 1999
7	Search for themes across dataset	Fluid	Braun and Clarke, 2006
8	Assign extracts to themes	Fluid	Braun and Clarke, 2006
9	Review themes	Fluid	Braun and Clarke, 2006
10	Define and name themes	Fluid	Braun and Clarke, 2006
11	Auditing of thematic material	Set	Influenced by Boyatzis, 1998; Elliot et al., 1999
12	Formally apply themes to research question	Set	*Developed in situ*
13	Thematic literature review	Fluid	Influenced by Charmaz, 2006
14	Thematic refinement	Fluid	*Developed in situ*
15	Write up findings	Fluid	Braun and Clarke, 2006; Charmaz, 2006

*This table illustrates the study’s adaptive thematic analytical procedure. The first author designed it in order to fit the research question and the shape of the data that resulted from the data collection process. The procedure was derived from two approaches to thematic analysis (Boyatzis, 1998; Braun and Clarke, 2006) and is influenced by Grounded Theory (e.g., Charmaz, 2006), anthropological analysis (emic and etic distinctions; Harris, 1968), guidelines for evaluating qualitative research (Elliot et al., 1999), and innovations as necessary (steps 5, 12, and 14). The ‘level of rigidity’ refers to the degree to which interaction can occur between steps: ‘fluid’ indicates give and take between adjacent steps, and ‘set’ indicates a fixed point.*

**TABLE 2:** Ethnographic practice and analysis procedure.

**Step number**	**Analytical stage**	**Example**
**Ongoing throughout project**	**Explore situation and context**	Approach site as a situated social practice
	Investigate systems *(e.g., procedure)*	Observed airport checkpoints during personal travel
	Examine structure *(e.g., technology)*	Analyzed body scanners from a design perspective
	Situate site in the social world *(e.g., current events, social discourse)*	Read (and consume other media) widely and reflected
	Challenge developing analysis *(e.g., member checks)*	Asked participants whether the analysis lines up with how they understand their own experience
1	**Theory-based coding**	Coded for recognition/misrecognition experiences
2	**Explore recognition/misrecognition extracts**	
	Code self-definition categories	e.g., Not a bad person; e.g., Uniformed government employee
	Code for how (mis)recognition is experienced	e.g., “Best transit ever” (recognition) e.g., “The worst experience” (misrecognition)
	Code for actions	e.g., Reproduction (recognition) e.g., Accommodation (misrecognition)
3	**Analyze recognition/misrecognition extracts for context**	
	Refer back to interviews/diaries	Situate extracts within participant’s other data
	Re-contact participants with follow-up questions	Situate extracts within new data
	Refer back to airport security context analysis	Did this experience take place before or after body scanners were introduced?
4	**Develop analytical structure**	Create initial analytical outline in which to write the analysis
5	**Challenge analytical structure**	
	Look for divergent cases	Look for conformity/non-conformity Search for exceptions
	Validate analysis by inviting critique	First and second author critically challenge analysis in conversation
6	**Confirm analytical structure**	Formalize the analytical outline
7	**Write analysis**	Communicate the analysis analytically and esthetically

*This table illustrates the ethnographic practice and analytical procedure designed by the first author. It was fluid and should not be read as a rigid list of isolated steps. The analysis involved exploring airport security as a social practice (the system, the structure, the situation). It also included theory-based coding for recognition/misrecognition experiences, an exploration of the recognition/misrecognition extracts (coded for self-definitions, coded for how recognition/misrecognition is experienced, coded for actions) and a contextual analysis (referring back to other data where, re-contacting participants where needed, and referring back to the airport security context analysis). Following that, the analytical structure was developed and challenged. Finally, the analytical structure was confirmed and the analysis was written, with attention paid to both analytical and aesthetic concerns.*

### Analytical Quality

Qualitative analysis validation is a contentious topic (see for instance, Hammersley, 2008). Our approach is that the research method, including the validity checks, should fit the needs of the research question, data, and study context. In this case, both authors are study population members (e.g., air travelers), and the project was initially very open. Therefore two colleagues audited a portion of these data (Elliot et al., 1999). One analyzed three of the initial interviews (*N* = 12), and another analyzed 10 of the initial diaries (*N* = 101) and all the thematic codes for those data. The interviews and diaries were all randomly selected, except in the case of the 2010 interviews, wherein one was specifically included due to the second coder’s expertise in disability studies.^4^ In most cases, everyone independently agreed; where analyses diverged, we then converged on a common analysis through critical discussion.

During the main analysis, the first author kept an eye toward divergent cases (e.g., checking for data that did not fit the developing analytical framework) and cultivated theoretical sensitivity, meaning she immersed herself in the topic and questioned her assumptions. For example, she interviewed a former airport dispatcher, examined media (e.g., news, film, literature) that referenced airport screening, observed airport checkpoints during travel, researched airport security laws, procedure, and technology, and explored other forms of surveillance (e.g., biometrics, data mining, and social network analysis) and other surveillance experiences shared by people who knew she was a surveillance-interested researcher (e.g., activist observations about riot police; experiences at national borders; the visa application process). She also discussed the developing analysis with the second author, accessible participants, and other interested parties (e.g., colleagues, conference audiences, friends, talkative strangers on airplanes) in order to challenge findings in terms of both theory and experience. In this way, the analysis should be read as deliberately collaborative.

Finally, both authors experienced airport security as travelers, observed it analytically as researchers, and at turns, experienced it from both perspectives at once. We aimed to make the impact of our status as air travelers an analytical strength. With regard to our positionality, that is, the way in which our own characteristics might impact the analysis, all we knew for sure in advance was the fact that our membership in the population of air travelers could either be a strength (e.g., opportunity for observation), or a weakness (e.g., risk of being blinded by assumptions). Therefore, the first author recorded and reflected on her own airport security experiences for 1 year (*N* = 8). She used those observations, and the observations of others (including the second author), to field-test the emerging analysis.

## Analysis

This analysis is divided into two parts. The first deals with recognition experiences, and the second deals with misrecognition experiences. In each part, the following questions are addressed:

(1) The different forms the concept takes;
(2) The ways recognition/misrecognition are experienced;
(3) The forms of action that occurred after these experiences.

### Recognition

#### Forms of Recognition

Participants described two distinct forms of recognition. In one, the traveler expressed a sense of being recognized in the screening process and mentioned that screeners openly communicated recognition (explicit recognition). In the other, the traveler expressed a sense of being recognized in the screening process, without indicating that screeners explicitly communicated recognition (implicit recognition).

##### Explicit recognition

In explicit recognition, the screener sees travelers as they see themselves, and communicates this. Here are two manifestations: mutual acceptance of a shared social identity and mutual inclusion within a shared, common ingroup. A clear example of both concerns military personnel traveling in uniform.

**FIGURE 1:**
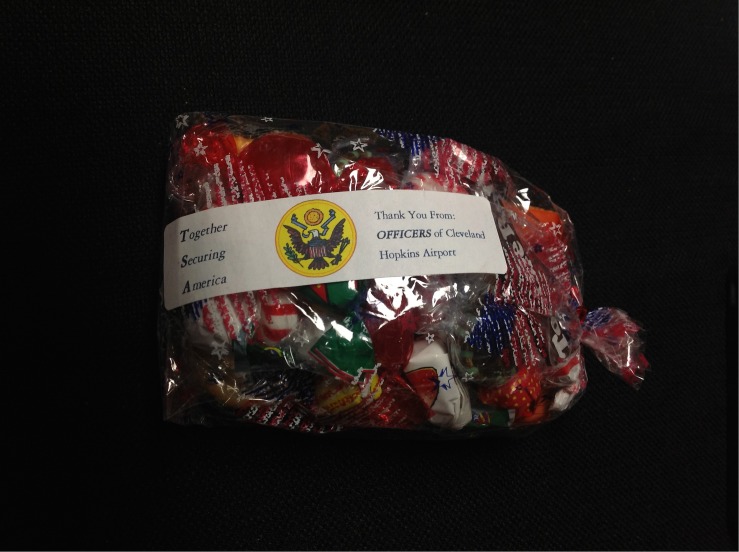
Gift given to George by TSA personnel. This is a photo of the snack gift George received from screeners. He photographed it himself, and shared it unprompted, along with his airport security diaries. It says (left) “***T****ogether* ***S****ecuring* ***A****merica”* and (right) “Thank You From: ***OFFICERS*** of Cleveland Hopkins Airport.” The center image is the United States’ coat of arms.

George (60M American) traveled through Cleveland Hopkins Airport (United States) in 2012. He reported a “very short wait to get to screeners – Went through the “First Class”/“Frequent Flyer” line as I was traveling in US Army military uniform. Gatekeeper TSA agent was warm and friendly and engaged me in brief conversation as she checked my military ID.…The agents did open my backpack after it passed through the X ray *[sic]* machine but notified me before doing so and were apologetic afterward. As I was waiting for the backpack to be cleared one of the TSA Agents approached me, thanked me for my service, and gave me a small gift pack of candy and crackers (photo attached).” The gift label said: *“****T****ogether* ***S****ecuring* ***A****merica”* and “Thank You From: ***OFFICERS*** of Cleveland Hopkins Airport,” and included the coat of arms of the United States (Figure 1).

George’s account and the snack gift show these TSA employees include themselves and military members together in a single group. These airport screeners construct the boundaries around the ‘group of people who secure America’ such that they include US military members (given that the label’s center image is the United States’ coat of arms), the Transportation Security Officers of Cleveland Hopkins Airport specifically, and the TSA as a whole (per the boldface text on the image’s left-hand side). What is more, they underscore their security-focused ingroup through their emphasis of the word ‘officers,’ which is an example of personnel terminology that is common both to the US military and the TSA.

However, shared group membership claims, once issued, may be accepted or rejected. In this case, George reciprocates, and also groups himself and screeners together; specifically, as ‘uniformed government employees.’ This is congruent with the screeners’ own ingroup category: these transportation security officers, and the military members who accept their shared group claim, are ***T****ogether* ***S****ecuring* ***A****merica.* Both the screeners and George structure their occupational self-understanding around keeping the surveillance-protected ingroup safe. The specific threat was not made explicit because it is understood, or expected to be understood, through shared sociocultural knowledge (see also, Jovchelovitch, 2007).

##### Implicit recognition

While explicit recognition is characterized by reciprocity, implicit recognition involves projection. At airport security, travelers who experience implicit recognition consider their own self-definitions consonant with the categories used within the screening process. This is well-expressed by Catherine (56F, American), who said that whenever she feels inconvenienced, or has to wait in line, she reminds herself that the screeners are just doing their job. She said this “helps me relate to them better. I think most people have had to do things they didn’t necessarily want to do, or enjoy doing, because of a job. I also realize that what they’re doing is considered necessary and designed to keep people, including those inconvenienced, safe.”

Here, Catherine uses two types of sense-making that are germane to implicit recognition. First, she indicates screeners are included within the group of ‘most people’ who have had to do work they do not enjoy. This is a group to which she herself feels she belongs, and that, in her thinking, includes most people. Second, screeners are a part of the security apparatus, and Catherine considers their work necessary. It has a purpose, and that purpose is safety. The implied threat, while undefined, exists somewhere outside of the ingroup. Catherine implicitly includes herself amongst the people kept safe by surveillance.

Irina (26F, Latvian) does as well. She transited through an airport checkpoint in England while on a business trip. “I feel myself normal about it,” she said: “Because this is them work and this doings for our security.” The common, safe ‘us’ is not nationally bounded here, as it was for the screeners George encountered while in his US Army uniform. However, Irina does speak more specifically of the threatening outgroup. Irina agrees that the British airport security officers “have to check people that possibilities for the terrorism become less.” She makes the threat clear, but still does not define who is included the outgoup, beyond implied terrorists. Implicit recognition as a part of a common ingroup is an affirmation that one is *not* a dangerous other, whomever they might be.

The account of Minh Tinh (66M, American), a Buddhist monk, demonstrates this evocatively. He also sees himself as part of the ‘common us’ along with the screeners, but he acknowledges that this may not be immediately apparent to security personnel. As Minh Tinh said, “I do dress unusually. I wear a long, brown robe over a shorter robe,” he said, “and my head is shaved, and yeah, I’m over six foot, and I’m a big man. So, physically, I can see how people would be apprehensive. And certainly I fit outside the mold.…So, prior to the TSA pre-check, I was always searched every time, my genitals felt and squeezed, and I had my butt felt and squeezed, and I was not particularly happy to have those intimate body parts touched in security.”

But nevertheless Minh Tinh endorses this as part of realizing recognition, and he retains a highly positive view of the screeners as doing a difficult job to protect the common ingroup. “At the same time,” he said, “if it keeps us safer, I would certainly be willing to walk through the thing butt naked. I have no great modesty. But I just thought it was– My genitals are sort of private, and I prefer strangers not fondle them…. [But] if it does keep us safe, I’m not unwilling to go through that again.”

The crucial term here is the ‘us’ in the last sentence. What Minh Tinh endures as an individual, he does for the benefit of others as a group member. This affirmative group relationship is reflected in his positive view of the screeners: “they,” he said, “the TSA, most of the staff, I believe, actually want to be friendly and kind, and people generally have been very nice and very pleasant to me. I’ve never seen anyone with an attitude. Even the few times where I’ve been touched very intimately, the person was like, ‘I’m sorry about that. We just have to make sure everything is safe.”’

The key element in implicit recognition is that the traveler feels screening is designed *for* people like them. In so far as there are others who want to attack ‘us’ and it is not clear who ‘they’ are, then everyone must be treated as suspicious in order to differentiate between the two. Implicit recognition is bound up with seeing the screening as a necessary part of incorporation to establish the traveler’s identity as ‘one of us.’ Here, it is a form of recognition in which travelers establish ingroup status with screeners, surveillance, and society as they exist together.

#### Experiences of Recognition

##### Explicit recognition

When asked how he felt about his military service-member recognition screening experience, George said, “Very Good. *[sic]* Best transit ever. Officers were warm and engaging. Part of this was my traveling in uniform, but I have traveled in uniform before when TSA was cold and unpleasant.…[This time], I felt appreciated, connected and had a good feeling ‘high.”’

This was not just a matter of having been given a gift (refer to Figure 1). “Remember,” George said, “the gift came at the end…. they were positive and engaging from the point of entry in the line, when my ticket was checked, before reaching the conveyor to the scanner. The gift at the end was like the crescendo of a good experience.”^5^

The gift, George said, “was an unexpected, unsolicited and free expression of goodwill on the part of the TSA staff. Unlike some gifts (for example to politicians, where there is an implied expectation of reciprocity at some point in the future) there could not be any explanation other than goodwill. That made it especially meaningful. The cynic might say that it was merely part of a bureaucratic scheme to curry favor with the public (I have know *[sic]* way to know the origin of the gift), but the way that it was delivered: personally, with eye contact and obvious emotional connection made the expression very meaningful, and memorable, for me.” The gift itself was symbolic, and made significant through George’s sense of connection with the security screeners in that moment.

George said that his positive experience was due to the recognition of his identity as a government employee and his common identity with the security personnel. Indeed, George actually uses the term recognition to explain the positivity of his experience. He said it had “to do with a recognition on the part of the TSA personnel, that they are, you know, kind of, uniformed government employees, and I’m a uniformed government employee.” And, as it is clear from what is written on the gift in the photograph (refer to Figure 1), this social identity indicates inclusion within a common, in this case national, ingroup.

##### Implicit recognition

Perhaps the most remarkable aspect of Minh Tinh’s account is the way in which he reacts to what, in another context, would be sexual assault. While he doesn’t express positive sentiments, neither does he express a strongly negative sentiment. Rather, his account is sardonic and mild: “my genitals are *sort of* private, and I *prefer* strangers not fondle them” [emphasis added]. What is more, in overall terms, despite their intrusions, Minh Tinh retains a positive view of the screening staff. “We’re all human beings on the same journey,” he said, “and in this process, it’s so easy to look at everyone as our brother and sister.”

Minh Tinh considers screeners to be ingroup members; therefore they are less ‘other.’ As a result, their presence and intimacy become less intrusive. Where screeners are not construed in this way, even mild intrusions are experienced much more negatively. Take Maria (30F, Greek/British), for instance, who positions the security staff as strangers. She recounts how having her bag searched made her feel “generally uneasy, despite the fact that I had nothing to hide and nothing illegal. Probably uncomfortable due to a sense that my privacy was being invaded by strangers peering into my bags.”

While some, like Maria, found airport surveillance uncomfortable, this scrutiny is a necessary precursor to implicit recognition. However onerous it might seem, it confirms ingroup membership. It follows from this that a lack of scrutiny might be more problematic than scrutiny. Without scrutiny, would it be difficult to discriminate ‘safe’ ingroup members from ‘dangerous’ outgroup members?

This is precisely what the findings show. Christiane (38F, German) complains about security staff who “didn’t seem particularly interested in the x-ray *[sic]* screen or the scanner or the passengers for that matter (their mobile phones seemed to require all their attention).” Anne (55F, British) concurs. She feels “that if they are going to search you they should do it in detail.” Trivial scrutiny that does not evoke recognition can be resented more than extreme scrutiny that does induce recognition.

All in all, recognition in both forms had a positive impact on experience. Explicit recognition created a highly positive experience of screening. Implicit recognition attenuated the negativity of scrutiny that might be invasive in other circumstances.

#### Reactions to Recognition

At one level, it is arguable that recognition, either explicit or implicit, invoked no particular reaction. Neither Minh Tinh nor George, both of whom the analysis followed in some detail, did anything different after recognition. They did not report any changes to their actions after experiencing recognition. But the argument that recognition creates no reaction misses the point: while consequences normally mean change in psychology, reproduction is a meaningful response to a social encounter.

The fact that these travelers would continue to go through security without changing their behavior in any particular way is significant given that, at least in the case of Minh Tinh, the way he was treated might, in other circumstances, lead one to expect some change. Remember, security screeners repeatedly touched intimate parts of his body. Minh Tinh didn’t like that, and wouldn’t accept this treatment from a stranger on the street. On the street, this would be an assault. At the airport, it was experienced in terms of ‘our’ safety. It was for the ‘common us,’ which made it bearable.

So, there is more to recognition than unchanged behavior. Both respondents not only indicated a willingness to go through airport security again, they would willingly do more. George in particular emphasized this point. “I did not feel like I was an anonymous frammet on an assembly line being processed: powerless, preyed upon. I felt more like a willing participant for the common good.…The attitude of the entire team made me happy to willfully engage. If they had wanted to turn my socks inside out, I would have been ok.”

The key point here is that George did not experience that security encounter as coercive or imposed. His actions were not mere compliance to an other’s will. They were an expression of what he wanted to do. If anything, after recognition, he was willing to accept more scrutiny. Like with Minh Tinh’s experience, context is critical. George accepts scrutiny at the airport for a common ingroup’s safety. While strangers invaded Maria’s bags, ingroup members opened George’s backpack. Scrutiny for the ingroup is, in George’s words, “ok.” Like Minh Tinh, he willfully engaged for the common good.

But this is not all. Implicit recognition not only reincorporated travelers into a ‘safe’ ingroup, it facilitated category transition. Having experienced screening as a positive affirmation rather than an imposition, George was motivated to act in similarly supportive ways toward others.

“The encounter,” he said, “‘made my day,’ and I felt like passing it on to someone else, in some way ‘paying it forward’ to another traveler. Frankly, the gift was a token that had little monetary value, but put me in a very good place for the rest of the day. I am sure that it positively affected the way that I interacted with other people for at least that time period.” Recognition not only encouraged travelers to keep doing the same thing, it also motivated participation and inspired a desire to incorporate others.

However, while recognition is associated with behavioral reproduction, engaged participation, reincorporation, and category transition, these are not given outcomes. First, one instance of recognition, even if profound, does not result in enduring participation. This is made clear when by returning to George’s observation that generally speaking, whether he travels in or out of uniform, he is “glad to get through without being hassled.” In fact, he usually tries to avoid unnecessarily engaging with screeners as a result of negative encounters, which is discussed with more depth in the *Reactions to Misrecognition* section.

Second, tensions arise when the recognized self-definition conflicts with an incongruent self-definition. Apple (26M, American) said, “I really think that the guards that are more alert, or more strict-faced, are simply doing their jobs. And, I accept that, as part of the national security.…Though, hopefully we will live in a world that won’t be so, you know, so rigid and cautious.”

Apple includes himself within a ‘common us’ like George, Catherine, Minh Tinh, and Irina. They all incorporate themselves into a surveillance-safe ingroup, and accept airport security on those grounds. As Apple’s account demonstrates, people have multiple self-definitions, some of which may come into conflict as they move through different contexts. Travelers can feel they are part of a surveillance-protected ingroup and experience that inclusion positively, but also reflect on that group membership from the position of a self-definition that has a bittersweet, not enthusiastic, acceptance of that ingroup status.

### Misrecognition

Misrecognition, like recognition, takes numerous forms. The analysis covers two types. The first, misrecognition by commission, occurs when one is defined as X by others or social practice, but X is contrary to self-definitions. This is illustrated by example with a specific subtype, threat misrecognition, which happens when people are positioned as dangerous even though they feel they are a part of a safe, ‘common us.’

The second form, misrecognition by omission, transpires when one is rejected as X, but X is consonant with self-definitions. This involves a sense that the screening process denies a self-definition, or denies its value. The examples demonstrate degraded national/racial categories and dehumanization. As with the recognition section, the analysis demonstrates different forms of misrecognition, shows how they are experienced, and finishes by discussing how people reacted.

#### Forms of Misrecognition

##### Misrecognition by commission

Sarah (60F, American) traveled to Texas to attend baseball games with her daughter in early June 2002, soon after “they first started with the heightened security stuff.” It was her first time flying since 9/11. Screeners searched their luggage each time they crossed a checkpoint. “It was just a pain in the behind,” she said, “because we had to stop and open our big suitcases every time.”

When asked to explain how she felt Sarah said, “nervous, I guess, would be a good way to describe it. Just wondering, you know, what’s going on. You know, ‘don’t look through my bags. I didn’t do anything.”’ Here, Sarah felt repeatedly categorized as a threat, when she categorizes herself as a non-threat. This was especially poignant because it was new experience for her. Being singled out felt personal. “Especially in the beginning of this, you felt more threatened personally, like they were singling you out as a bad person, that they needed to double check on.” Threat misrecognition personalizes surveillance. It makes it about scrutinizing you, rather than about protecting the ingroup.

One way of thinking about threat misrecognition is that it is akin to implicit recognition, without confidence in the reincorporation process. The focus is on the moment of mistrust and scrutiny, without the belief that it will eventually lead to one being correctly accepted as a safe and respectable ingroup member. To travelers who experience misrecognition by commission, it may seem that the screening process is unable to properly determine who is genuinely ingroup and who is not. Lorna’s (55F, British) account demonstrates this finding. She is a frequent business traveler, and prepares for screening to avoid problems.

“I am very willing to take off most of my outer clothes,” she said, “empty my bags and help the process along. I make time for all this when I am at an airport and I have learned what to do and try to dress appropriately so that the search and strip off is not too much of a hassle for me. Eg *[sic]* wear slip-on shoes, no belts, and carry no liquids.” In general, then, screening does not particularly bother Lorna. She accepts it as a way of protecting safety, even though she dislikes some aspects. “I do not like being frisked,” she said, “as it is a very close and personal search (busoms *[sic]*, hand down back of trousers, and crotch). Although I believe it important to be safe.” Most of her security experiences are, in her words, “pretty easy going, as I am so used to all of the security checks.”

On one occasion screeners delayed Lorna for about 5 minutes. “No real time lost,” she said. What did annoy her about this particular incident (“very much,” in her own words) was that in her eyes, it demonstrated inadequate procedure. It involved “a woman behind me dressed in full muslim *[sic]* dress/clothing and head gear.” The screeners conducted a body search on Lorna, but, she said, ignored the other woman.

“In my book,” Lorna said, “she would have been the very person I would have searched if I had been security. The volumes of clothing and masked face appeared to make a mockery of all the security and searches we have to endure! Maybe she was searched in another room, but I didn’t see it. I asked myself why didn’t the security team conduct a similar search on her? I was not impressed at all! Now that makes me nervous…Muslims not undergoing the same checks as everyone else.”^6^

Because screeners don’t scrutinize someone she views as suspect, Lorna concludes that the security process cannot efficiently sort people into ‘safe ingroup’ or ‘dangerous outgroup’ categories. Thus, screening has no reintegrative function. It becomes useless intrusion.

What is more, once Lorna feels people are scrutinized inconsistently, the choice of who to scrutinize is experienced as personal. It is a judgment of who is, and who is not, suspect. She thereby experiences it as misrecognition in both an absolute sense (why treat me as dangerous) and, even more acutely, in relative terms (why treat me as more dangerous than someone who [in her perspective] has far more reason to be placed in that category). Being treated as dangerous is not a necessary precursor for being recognized as safe. Instead, being treated as dangerous can misrecognize selfhood.

##### Misrecognition by omission

Vik (26M, Indian) is a graduate student studying in the United States. He catches at least two flights or more from his home base in India to his Midwestern university. “The worst experience I had was in London,” he said, referring to the time he transferred between flights soon after the liquid explosives plot that occurred in August 2006 (Chertoff et al., 2006). The airport strictly regulated all carry-on luggage at that time.

“Thing was,” he said, “at the Indian airport, we were not told those things would happen at the British airport. So, when we went to the London airport, they actually took…everybody’s cabin [luggage], and told it has to go to check-in. They gave everyone a small, thin bag; just put in your passport and documents, nothing else. I mean, you have some 300, 400 people, you give them something like that, the possibilities of losing stuff is pretty much high.”

“Luckily,” he continued, “at that time, I carried a very thin bag, I mean, from India itself…. I did read in the papers, so I had some idea. So, I didn’t have that much issue [with my own luggage].” Instead, Vik’s problem was with how the London airport security personnel treated him and his fellow Indian nationals. “They literally treated us like some– In a very bad way. I mean, not as like, I don’t know what to say, the word, I don’t want to use a bad word, but I just felt that we were very treated like trash.”

Vik also observed differences in how the airport treated Indian and European travelers. “Personally,” he said, “I didn’t feel like they really treated everyone– I don’t know, I mean, that’s, to me, it was kind of racist. In the [British] airport you also have the Europeans also. They were not treated the way the Indians were treated. That was something I was not happy with…. It was so blatant. I mean, you can never miss it; it was like that kind of a case. The way they were actually treating…. I mean, in US also we came [later that day], but yet nothing happened. Yet, also, they did check-up, but not to that extent. I mean, at least they treated us with some dignity. Which is important.”

Vik’s account has been explored in some detail because it contains two intermixed misrecognition by omission categories: that of his humanity and that of his nationality, which he communicated in intersectional terms with race. First, Vik objects to being treated as trash, as a worthless object. His humanity is ignored. He is not treated as a person with feelings and rights. He is thoroughly dehumanized. What is more, that dehumanization is selective. Vik is selectively treated badly as an Indian. Europeans are not treated in the same way. Vik’s national group membership as an Indian is defined negatively, and is positioned as a warrant that he (and his fellow Indians) do not deserve respectful treatment.

These two categories need not always be intermixed. Often people resent their humanity being ignored without ascribing their treatment to an inappropriate view of their group membership. For instance both Nora (25F, American) and Emily (24F, American) express concern at the ways in which they are treated as objects, open to the gaze of others with no human rights to privacy or dignity. Nora is concerned with body scanners. Who sees the scans? “Nobody I have authorized to see my body, not like in medicine where I can establish a relationship of trust.” Emily shares this concern, although she finds being patted down even worse: “I don’t like someone looking at me basically on display,” she said, “but I almost prefer it to having a stranger’s hands on me (for pat downs). Anyone can look at me and imagine me without clothes on and see my figure, but not everyone gets to touch (you know what I mean – the latter just feels more invasive).”

#### Experiences of Misrecognition

Both misrecognition by commission and omission resulted in strong negative emotions. In the case of threat misrecognition, a form of misrecognition by commission, travelers typically reported mixed and unsettled emotions. When Ellie (24F, American) visited England in 2008, a screener asked to look at her laptop.

“And, I got very nervous,” she said, “and very kind of, I didn’t know what was going on, and I obviously said yes, because if I said no, then they’d probably take me away, and it would be very bad.…When someone asks you that, you kind of go through your mind, ‘Well, did I touch something, did I do something,’ and it kind of goes through that thought process of ‘I hope I don’t get in trouble, I hope I didn’t touch something that I’ll get in trouble,’ that kind of thing. Not that I thought I did, but that thought process kind of goes through your head whenever someone grabs your stuff and says ‘Is that yours?’ And you’re like, ‘uh, yes? Maybe?”’

In the case of misrecognition by omission, there is similar negativity but less uncertainty. Nora rejected an international trip soon after airport security introduced body scanners. As a result, she said, “I have had to reflect, and articulate for myself why I had that reaction [to the body scanners]. If anything, it has made me more confident in what I am…. How the security is conducted does not respect who I am.”

Misrecognition by omission also invokes anger. Fiona (24F, British) described her experience at a Scottish airport: “As I passed through the detector, it bleeped and a woman who was talking to a colleague pulled me aside. She proceeded to rub down my arms, stomach, and legs while still talking to a colleague…. I think what annoyed me most of the whole experience was that the woman who searched me continued to talk whilst searching me. I found this very unprofessional and I felt as though I was in a ‘cattle market’ – just someone else to process, more hassle for them.”

#### Reactions to Misrecognition

##### Avoidance

The first way travelers react to misrecognition is simply to avoid the sites where it occurs. Sometimes this is a matter of avoiding particular airports where one has been misrecognized, or thinks misrecognition is likely. Recall how Vik experienced dehumanizing treatment in a London airport, linked to anti-Indian discrimination. He now tries to avoid that layover location: “Unless I go to London, I try not to connect to London.”

Some avoid aspects of the process itself. Nora, as demonstrated previously, objects to body scanners that remove her dignity and lay her open to the gaze of all. “It is not for strangers to see my body,” she said. “When I fly, I have always opted out of the scanner…. Usually I am traveling with an infant in arms, so I’ve gone through the metal detector.” However, without an infant to divert her to the metal detector, the only other option is a thorough pat-down. Nora is equally unwilling to be frisked, and she explained why. ‘Traveling with children, it’s difficult for children psychologically to see police/authorities scrutinizing parents.’^7^ Each time she books a flight, it’s very stressful wondering whether she’ll be able to go through the metal detector or not.

Sometimes, where the misrecognition experience is linked less to a specific site or procedure, and more to generic aspects of the screening process, the reaction is to stop flying altogether. Charlotte (23F, British) expressed a fear of screening because she always anticipates that some valued part of her self will be denied or rejected. She no longer travels by air, even though this comes at a cost. As she observed, ‘I think I’m just punishing myself by avoiding, probably. But I feel like I’m resisting.’^8^ Charlotte refuses to be miscategorized by airport security, and as a result, is unable to travel by air.

##### Accommodation

A second way in which people respond to misrecognition experiences is to change their behavior to adapt to the operation of the security system, and thereby make themselves less likely to be picked out and exposed to special scrutiny. Remember how Sarah, who traveled with her daughter to attend baseball games, experienced threat misrecognition as she was repeatedly stopped and searched at security checkpoints. Eventually, she discovered the problem.

“I had brought newspapers to keep the box scores for the ballgames we went to,” she said. “And I wanted to bring that stuff home with me. So I stuck the newspapers…in the front pockets. And when they lay the suitcase on its back, and shine the X-ray through it, they can’t get through the newspapers. So they opened it up.” So Sarah adapted her behavior. “From that point on, I have always put any kind of papers on the bottom of the bag. I made sure…. And, I’ve never had a problem.”

George, whose recognition experience was discussed with some depth earlier, also experienced problems with a luggage search. However, while being positioned as a threat preceded Sarah’s misrecognition, George’s misrecognition was due to a lack of respect. “I’ve gone through security, and for whatever reason, they want to visually open and inspect the bag,” he said. “I asked what the issue was. They did not tell me. I suspected it may be a camera, a camera lens, because that can be very dense, and perhaps confusing on the X-ray, on the radiographic screening that they do. So, I offered to show them exactly where the camera was, so that they wouldn’t have to root through my bag. Save them time, and save me intrusion. And, they were very curt, to the point of being unpleasant, and told me that I was to back away and not touch any of the bags, and basically be quiet. And, I said, ‘you know, I was just trying to help.’ And, I was not impressed with their level of professionalism or appropriateness.”

So George, like Sarah, adapted his behavior. He carefully arranges his bags to avoid attracting attention. “I minimize anything that would be a question or a distraction,” he said “particularly in the packed bags. I don’t put anything in them that would be so radiopaque, or difficult to X-Ray, that they can’t tell obviously what it is. So, for example, with a camera, I don’t leave it in the bag anymore. I will have it on top of my suit coat and shoes, so that it’s easily visualized and seen. I also ensure that as it’s sitting on the belt, that it goes through in such a way as to be easier to appreciate what it is…. Number two is that I don’t even speak to them. I don’t engage them. They ask a question, I give a short answer. They don’t ask a question, I don’t say anything.” This allows him to avoid having any “personal interactions with any of the folks who are working as security screeners.”

##### Resistance

Two “quite polite employees” stopped Kostas (25M, Greek) just before he went into the security checkpoint. They “made me drink the entire bottle of cola before proceeding,” he said. “Technically, I didn’t have to drink it, just throw it away, but I did enjoy their awkwardness when watching me drink slowly.”

Nora, who finds frisking and body scans adverse, also commented on screener interaction. “I try to make it more personal,” she said. “Like, if the questions are being rattled off, I may like, hesitate, and then like, actually make eye contact with the person before I respond to the question, just to kind of, like, break the routine, so to speak, to kind of recognize, like pause and be like hey, like, ‘it’s not just– it’s not just dealing with goods, passing through, or these toxic goods or these non-toxic goods,’ but that it’s an actual person, interpersonal interaction, albeit perfect strangers.” Nora wants to be treated like a human being, not a commodity, so she subtly changes the communication dynamic between herself and screeners.

Both of these examples can be seen as instances of resistance. They involve attempts to regain a degree of agency and to acquire some control in the screening process. To some extent they may be seen as successful: Kostas makes the security personnel wait for him and Nora makes them acknowledge her human presence. At the same time they are very constrained and partial victories. Kostas might drink his soda slowly but he is still forced to consume his drink rather than take it with him. Nora might make eye contact, but is still subject to processing she finds objectionable. Ultimately, these travelers can only change how they comply with security demands, not whether they comply.

The reason is obvious. If resistance goes too far, the security staff has the ability to forbid air travel, or even worse. As Andrea (29F, Chilean) said, “you can get really anxious. Not because of they treating you bad, because you feel that you can eventually get in a unpleasant situation.”

Even watching resistance evokes anxiety because of the potential consequences. Louise (28F, Irish) observed a disagreement between a traveler and screening staff in a British airport. She “noticed a young man questioning his bag being searched – two members of airport security were with him, one seemed to be the other’s supervisor. I wished the young traveler would stop questioning as it was making the staff very defensive.” Here, the staff’s defensiveness is the patina on the surface of an embedded risk: travelers may be sorted out of the ‘safe us’ and into a ‘dangerous other’ at the discretion of surveillers.

## Discussion

The analysis addressed three questions:

(1) What are the forms of recognition and misrecognition that people experience at airports?
(2) How are these experienced?
(3) How do people react?

The *Surveillance Experiences and the (Mis)recognized Self* section summarizes and contextualizes the answers to these questions. Then, as promised the *Autonomy, the Variable Self, and Context* section uses these findings to explore the implications of the (mis)recognized self for autonomy.

### Surveillance Experiences and the (Mis)recognized Self

#### On Recognition

The analysis explored two forms of recognition (explicit and implicit) that occurred at airports as a result of security screening. It demonstrated *explicit recognition* with an incident where airport screeners claimed shared group membership with a military officer, and the officer in turn accepted this claim. Here, the screeners affirmed a valued social identity. This recognition was mutual; the military officer in turn saw the screeners as uniformed government employees, along with himself. Within this case, screeners referenced a safe ingroup (see Figure 1), to which they included military members generally, and George specifically, through their actions. This illustrates some of the characteristics these screeners used to determine their own group boundaries, which they then subsequently recognized in George.

In *implicit recognition*, airport surveillance itself served as a recognition vector. This occurred when travelers projected a relatable category onto an aspect of the surveillance process; here, the example was ‘people who have had to do work they don’t enjoy.’ Another form of implicit recognition occurred when travelers felt included within a ‘common us,’ which was understood as an airport-security protected safe ingroup. In this case, travelers projected their own sense that airport security exists to protect ‘people like them’ onto airport screening, and felt recognized as a result. The process of airport security physically separates people, processes them, and then recognition psychologically reintegrates them into the ‘safe us.’

Whichever its form, recognition improves security screening experiences. This can be a matter of making experiences that might ordinarily be invasive and unpleasant, such as intimate body searches, less negative. In other cases it transforms screening into an experience that is memorable for its extreme positivity. Because of this improved experience, recognition does not put travelers off the screening process. It does not lead them to change their behavior in order to change their experience. If anything the contrary is true. Recognition led travelers not only to accept screening, but also to be open to more. Recall, for instance, George’s remark that recognition made his day. In that moment, if screeners had asked, he would have been okay with turning his socks inside out.

This response to recognition looks like engaged followership, a type of identification that occurs when people identify with leaders and the community they represent (Reicher et al., 2012). Here, instead of working toward a leader’s explicit goals, the traveler participates, along with screeners and the surveillance apparatus, toward the ingroup community’s socioculturally bounded goals: safety for the ‘common us.’ This is engaged participation, as it were, rather than submission to authority or grudging acquiescence. Therefore, it should be no surprise that travelers are amenable to accepting airport security when they experience recognition during screening. In fact, this finding is akin to closed circuit television (CCTV) studies that found shared identity results in more surveillance acceptance (O’Donnell et al., 2010a, b), and a study of online surveillance that indicated participants objected when they felt surveillance misrepresented them (Stuart and Levine, 2017).

This is not to say recognition gives carte blanche for surveillance. One can feel fully recognized as a member of a ‘safe’ ingroup, but simultaneously express qualified acceptance when recognition on one self-definition occurs at the same time as misrecognition on another self-definition. Recall how Apple endorsed airport surveillance as something that exists for ‘our safety,’ but also expressed regret and wished for a less rigid and cautious society. The present study’s findings, in conjunction with research that showed qualified support for CCTV in public space (Dixon et al., 2003), suggests this reaction may be a more a response to being categorized and scrutinized, rather than related to the specific surveillance modality, like airport security or CCTV. Taken together, this suggests that friction between self-definitions is implicated in qualified support for surveillance; future studies can test this hypothesis.

Finally, this study shows recognition has the power to move people from one category to another, a phenomena which the psychology literature has termed ‘recategorization.’ What this means, in plain terms, is category transition. Recall that George doesn’t always have good experiences with screeners. Sometimes, he positions them as outgroup members due to being unimpressed with the TSA’s “level of professionalism [and] appropriateness.” In the present study, George reframed this group (TSA officers) after they showed him they were ‘recognizable’ as members of a common ingroup. A similar finding has been demonstrated in experiments (Simon et al., 2015). The present study takes this one step further by showing implicit recognition is implicated in self-definition transition. Future studies can explore the circumstances under which this is (or is not) the case.

#### On Misrecognition

When it comes to misrecognition, things are very different. *Misrecognition by commission* occurred when hetero self-definitions (that is, imposed self-definitions) were dissonant with auto self-definitions. Threat misrecognition, a specific subtype of misrecognition by commission, occurred when travelers who identified as unthreatening members of the surveillance-protected ingroup instead felt airport security treated them as personally dangerous.

Threat misrecognition can come from a sense that screening procedures are flawed, and thus unable to determine who is ‘safe’ and who is ‘dangerous.’ Once confidence in reincorporation into the ‘safe us’ is lost, then the mere fact of being scrutinized becomes contested. Surveillance is pointless and intrusive when it is not for ‘our’ safety.

*Misrecognition by omission*, on the other hand, refers to instances where valued identities are denied or misperceived. In the case of devalued national/racial categories and dehumanization, either one’s identity as a human being, with attendant rights and dignity, is violated, or else a specific group membership (in Vik’s case, as Indian) is treated negatively. The separation here is analytic rather than substantive. Often the two are intertwined: Vik is treated as trash because he is viewed as Indian.

With regard to experience, misrecognition is the opposite of recognition. It is experienced negatively: a combination of unwelcome confusion (‘why am I picked on, what did I do’), distress, and anger. In recognition, ingroup members open bags. In misrecognition, strangers invade them. What is acceptable in one context becomes intrusive in another. In recognition, acts that would be otherwise invasive can be experienced as mildly discomforting, or even actively endorsed. The opposite is the case with misrecognition: what might be minor intrusion in another circumstance is strongly discomforting.

Such is the negativity of misrecognition experiences that the sampled travelers changed their behavior, going to considerable lengths to try and ensure these experiences don’t reoccur. Travelers try to avoid sites where they experienced misrecognition, because they anticipate it happening again, even to the extent of eschewing air travel entirely. If they continue to travel through potential misrecognition sites, they take great care not to do things that might invoke scrutiny. Or, they try to reassert a measure of agency and dignity, but are limited by the knowledge that if they confront authorities directly, they will be the ones to lose.

So, resistance is risky, and thus limited; however, this does not mean these travelers accepted imposed self-definitions. The people in this sample are very clear about who they are. They are apt to question the system after misrecognition rather than question themselves. As Nora reported, misrecognition inspired confidence in her own self-definitions. But, this may not always be the case. For instance, this study showed imposed categories stimulated avoidance, accommodation, and resistance. None of these involved category acceptance, though this has been demonstrated in other studies (Moss and Vollhardt, 2016). Clearly, some people do adopt imposed categories, though as yet this topic remains under-researched.

Finally, in the present study, participants experienced recognition as a positive affirmation. But what happens when people accept recognized self-definitions, but don’t want to be read on that dimension in a given temporospatial context? Recognition functions as misrecognition when it coincides with a desire to be read on a different self-definition (McNamara, 2019). A clear example can be seen in the case of military members and veterans. George was quite happy to be thanked for his service during screening; in his case, he experienced something like the collective effervescence (e.g., Olaveson, 2001) reported during positively experienced crowd events (Hopkins et al., 2016). But there is reason to believe recognition is not uniformly positive or desirable. It can be unwanted and experienced negatively; in which case, it is no longer truly recognition (McNamara, 2019).

#### Strengths, Limitations, and Pushing Toward Future Research

First, while some research suggests that interviews and diaries result in different types of information that might not be comparable in one analysis (Bornat and Bytheway, 2012), this is not the case in the present study. The difference is likely due to the fact Bornat and Bytheway (2012) were interested in life histories. Their observations suggest it may be problematic to uncritically combine long-term reflection and reflection on everyday life. The present paper’s analytical focus, however, falls into the realm of ‘everyday life,’ with some longitudinal overlap in terms of how people contextualized their own experiences.

While some participants did contextualize their experience temporally, these types of reflections were present, unprompted, in both the retrospective interviews and diaries completed soon after travel.^9^ Therefore, unlike Bornat and Bytheway (2012), the present study’s interviews and diaries resulted in analytically comparable data, so there was no logical problem with approaching these samples as one dataset. And, in fact, the combined dataset was a strength of this study, as the first author avoided over-sampling memorable outliers (a limitation of retrospective interviews) and effectively sought non-conforming cases (a limitation of pre-travel recruitment diaries).

Second, readers might ask whether there were differences between responses in the diaries and interviews that impacted the analysis. There were no analytically relevant differences in richness between datasets (when richness is defined as details necessary for conducting the analysis) except in the case of two diary respondents who did not answer the reflection question (e.g., how did you feel about this). As explained in the methods section, these were excluded as data because those respondents opted out of future contact.

It is, however, worth mentioning that some participants had a first language other than English, and the first author asked all questions in English. If this paper reported a study specifically on emotional complexity (e.g., Grossmann et al., 2016), or a study on culturally bounded phenomena (e.g., Nogueira et al., 2015), rather than an exploratory study on recognition/misrecognition during airport screening, then this approach might be a flaw. But those were not the questions answered in this paper, and all participants (sans the excluded two) responded with a level of detail sufficient for the analysis. Future studies can investigate culture, emotion and recognition/misrecognition specifically and use questions in local languages where appropriate.

Third, when the first author was preparing this manuscript, a colleague asked her whether she thought all of the requested demographic categories were relevant to the surveillance experience analysis. Simply put, not necessarily. This paper’s analysis was oriented around the categories participants raised in their screening accounts. So the demographic categories should be understood as relevant to describing the sample as a whole, not necessarily as relevant to the individual accounts. The demographic categories were chosen in order to describe an international, mixed student/non-student sample to readers with enough information so as paint a picture, but not so much that the categories risked priming participants or asking them unnecessarily intimate questions.

For instance, participants weren’t asked to report past sexual trauma, which can make pat-downs difficult (e.g., Dailey, 2010). There is, after all, no reason to assume that when a category is relevant to experience in the past, it will always be relevant in the future. This is in line with the philosophical basis of this study: an intent to be open to unknown unknowns, such as the relevance of ‘threat misrecognition’ or ‘recognition as a member of a common ingroup’ to how otherwise privileged groups experience surveillance. Seeing these concepts situated in rich, contextual data made their relevance clear. This approach was also integral to discovering and theorizing implicit recognition.

Fourth, while these data were drawn from a mixed student/non-student sample (a particular strength of this study), the sample skewed toward highly educated white Americans and Western Europeans. This reflects the university populations the first author had access to during data collection, and it also reflects the fact that the accessible people who had time to be gatekeepers were themselves privileged with enough free time (time wealth; Chatzitheochari and Arber, 2012) to hand out and collect diaries, or do the groundwork to find interviewees. Future research on recognition/misrecognition should intentionally seek wider representation.

Fifth, even though the study was designed around an open perspective, intended to maximize the space participants had to discuss their own concerns rather than the presumed concerns generated by researchers, these participants may have instead only raised topics that they assumed were of interest to the first author. Therefore, this study should not be read as an exhaustive account of all of the possible types of recognition/misrecognition.

To conclude this section, future research should also investigate how recognition/misrecognition is (or is not) relevant to empirical topics other than surveillance. To start the process of pushing forward recognition/misrecognition research, the final portion of the paper presents a problematization of autonomy, selfhood, and context in light of the recognition/misrecognition and surveillance analysis.

### Autonomy, the Variable Self, and Context

The first section shows that autonomy needs a recognized self. The second section is a brief analysis of airport surveillance in terms of whether it is an autonomy supportive or coercive context.

#### Autonomy: The Freedom to Define Selfhood and Act From Self One Chooses

Let’s return once more to how autonomy is generally defined within the psychological literature. It involves self-rule, self-regulation, and willingly endorsed action (e.g., Ryan et al., 2015). By definition, autonomy requires the ability to act freely on the basis of ones own self.

This means autonomy comprises two freedoms, not just one. The first is the freedom to determine the self one acts upon. The second is the freedom to act on that self. This means that autonomy can be compromised at two levels. Either the subject can be constrained in terms of self-definition, or they can be constrained in terms of the ability to act on a chosen self-definition. This distinction may be obscured if selfhood is viewed as singular, or if the possibility of multiple selves is alluded to without attendance to its structure, function or context. However, the question of ‘choice of self’ becomes critical if we view selfhood as multiple and contextually variable. That is precisely what the variable self gives us, and why recognition and misrecognition are fundamental to autonomy.

Under conditions of recognition, even behaviors that might be seen as imposed (submitting to scrutiny), and therefore fall outside conventional autonomy definitions, are seen as acts of volition from the recognized actor’s perspective. Those who anticipated or experienced recognition wanted to be screened. They wanted to be screened thoroughly. They would choose to increase participation, should that choice be offered. For in complying in this way, their sense of self is affirmed. If autonomy is to be defined only in terms of volition, then they are acting autonomously, even within a context of power inequality and constraint.^10^

Misrecognition, however, denies people the self they would choose, and consequently the ability to act on that self. For the misrecognized, their preoccupation becomes to avoid further misrecognition, or else to recover their desired self. They avoid places they would otherwise want to go, and they go through elaborate procedures that they would not otherwise choose to undertake. In neither case could this be described as autonomous action. Resistance, one might think, would be different. Here people reassert who they are and act on their own autonomous terms. But this paper demonstrates how limited resistance can be under conditions of misrecognition.

In sum, none of the range of possibilities open to travelers under conditions of misrecognition are entirely satisfactory. They are all strategies to deal with a situation where one cannot act on the basis of the self one would choose, but none of them entirely restores that choice of selfhood. But, this is not all. There is still the issue of whether contexts are autonomy supportive or coercive.

#### Is Airport Surveillance Autonomy Supportive or Coercive?

As Martin (2010) observed, travelers are expected to make themselves legible to airport security. What this means is that they, like those in Eggers’ (2013) Silicon Valley surveillance culture narrative, are expected to be both transparent (totally open) and readable (totally understandable). They are expected to expose themselves to scrutiny (see also, Ball, 2009) in order to be allowed to fly. Analytically, this reads as a coercive context: either open yourself to surveillance, or don’t fly.

So, one might come to the conclusion that airport surveillance is incompatible with autonomy. In other words, perhaps the airport security checkpoint is simply a heteronomous context. But that would be missing the point. While Nora and Charlotte were unable to act from their chosen self-definitions and also expose themselves to airport surveillance’s gaze, others clearly demonstrated a willingness to expose more. They were engaged participants in surveillance because they identified with their surveillance-protected ingroup, and were recognized as members of this ingroup. As another example, Kostas gleefully resisted, suggesting misrecognition on one self-definition may, in some circumstances, open the possibility of autonomy on another self-definition.

So it is not a matter of whether surveillance is or is not an autonomy-supportive context in any absolute sense. The critical point is surveillance experience has a frame of reference; it is perceived from a position (see also, Haslam and Turner, 1995). And, this frame of reference is not static. It changes. Even with the same person, surveillance engagement differs. When George experiences recognition, he reports engagement. When he experiences misrecognition, he reports disengagement. So, recognition/misrecognition is implicated in how travelers perceive airport security, and how they act as a result. A context that is perceived as autonomy-supportive on one self-definition may well be heteronomous on another self-definition.

### Toward a Future Autonomy Research Agenda

The autonomy definition used within much of psychology is limited by its implicit construction of a single, unified self, whether this self is co-constructed by family and culture (e.g., Kağitçibaşi, 1996), is to some degree related (e.g., Ryan and Deci, 2000b), or separate (Deci and Ryan, 1985). None of these approaches integrate the way in which the variable self functions as a result of context, and none take into consideration the consequences of recognized or misrecognized selfhood for action. Therefore, this paper puts forward an autonomy framework that integrates a multiple, context-variable self and a more sensitive context definition.

But, the analysis is limited in terms of what it can say about *how* people experience autonomy. This is because the study design was not constructed with autonomy in mind. The autonomy analysis was a *post hoc* assessment. Future studies should be constructed around understanding how participants experience autonomy from their perspective. So, here are some questions to start a new autonomy research agenda:

1. Is being asked to act from a hetero-self-definition when you would prefer to act upon an auto-self definition experienced as autonomy or heteronomy?
2. What are the consequences for autonomy under circumstances where social practices prohibit people from acting on the self they prefer?

Since the autonomy literature assumes a single self, questions like these risk remaining unasked. If we assume selfhood, we risk misinterpreting the meaning of actions. To put it in more theoretically cogent terms, under-theorizing selfhood risks secondary phenomena (thought/action) masking primary phenomena (recognized/misrecognized selfhood).^11^ And from there, whether an action is autonomous or heteronomous becomes rather muddy.

This is not just a theory problem, however. It is also a practice problem. Misrecognition structurally limits the ability to act upon on auto self-definitions (McNamara, 2019; see also, Hopkins and Blackwood, 2011), which may lead to social withdrawal (Blackwood et al., 2015; McNamara, 2019). Therefore, we should also explore the consequences of interventions that are designed without a realistic understanding of selfhood.

## Conclusion

Self-definitions are fundamental to surveillance experience. When airport surveillance imposes self-definitions, they may be consonant or dissonant with how travelers define themselves. Recognition occurred when screeners affirmed travelers’ auto-self definitions *(explicit recognition)* or travelers projected recognition on the screening process *(implicit recognition).* The sampled travelers experienced recognition positively, except when recognition conflicted with a dissonant self-definition. They reproduced their behavior, reported willingness to undergo searches that would be considered invasive in other circumstances, incorporated themselves into a common ingroup, and incorporated others into a common ingroup. While recognized travelers were engaged security participants, misrecognized travelers withdrew, accommodated, or enacted limited resistance. Misrecognition occurred when screening imposed dissonant categories *(misrecognition by commission)* and when it devalued or denied auto self-definitions *(misrecognition by omission).* This was negatively experienced.

Finally, this paper theorized autonomy by problematizing the self and autonomy through the lens of previous analyses. With recognition, behaviors that might seem to be enforced by an outside authority are instead experienced as acts of volition. Misrecognition denied auto self-definitions, so actions then centered on avoiding misrecognition or asserting self-definitions. None of these actions, however, restored the choice of self, nor the ability to act on the self one chooses. Therefore, autonomy is conceptually limited when it does not integrate the variable self. What is more, autonomy is inhibited if people cannot choose the self they wish to act upon, or are constrained to act on a self they do not choose. The autonomous self is multiple; contextually variable, and acts within a supportive context.

In sum, recognition/misrecognition are central to surveillance experience, selfhood, and autonomy. Autonomy requires the freedom to choose selfhood, as well as the freedom to enact selfhood. Autonomy is inhibited if people cannot choose the self they wish to act upon, or are constrained to act on a self they do not choose. This has implications for how studies are designed, how findings are used to build theory, and how theory is used to design interventions.

## Ethics Statement

We obtained permission for this research from ethics committees at Cambridge and St Andrews.

## Author Contributions

MM designed the studies, collected the data, conducted the first and second analyses, discerned the autonomy theory analysis, composed drafts, wrote, revised, edited, and prepared the final manuscript, and reviewed both proofs. SR supervised the second analysis, commented on and contributed to the first draft, and reviewed the first proof.

## Conflict of Interest

The authors declare that the research was conducted in the absence of any commercial or financial relationships that could be construed as a potential conflict of interest.

## Footnotes

1 These contexts are myriad, including (but not limited to) ideological, social, structural (Reicher, 2004) and intersectional contexts (e.g., Stephens et al., 2012).

2 Seven of these participants qualified their categorization: White – Irish American (*N* = 1), White – British (*N* = 1), White – German (*N* = 1), White – Irish (*N* = 1), White – Latvian (*N* = 1), White – Non-Hispanic (*N* = 1), and White – Scottish (*N* = 1).

3 Two diaries were excluded from the analysis because these respondents (who contributed to the Latvian data collection) had “never traveled using airport service,” and provided no further information. There was no possibility of follow-up (both opted out of all future contact), so it was therefore not possible to analyze these data. As a result, these respondents were not counted as participants.

4 That interview included an account of undergoing screening in a wheelchair.

5 This quote was reported exactly as written: the participant used this ellipsis to indicate a pause.

6 This quote was reported exactly as written: the participant used this ellipsis to indicate a pause.

7 Quote from notes taken during an unrecorded interview.

8 Quote from notes taken immediately after an unrecorded interview.

9 *Interview example: “*When I think of my experiences I kind of think of the different changes of how I viewed security, as a very young traveler, and now as a more mature traveler (Nora, 25F, American).” *Diary example:* “Although I am used to the procedure, due to frequent travel, it is still baffling that this amount of security measures is used, even in non-international flights; it seems a bit paranoid, given the relative absense of highjacking since 2001 (Daniel, 25F, German/Cuban).”

10 To be clear: while the findings suggest that autonomy is *possible* with recognition during airport surveillance, there is nothing here that suggests that *all* behaviors that occur under conditions of recognition are autonomous.

11 McNamara, M. E. (manuscript in preparation). *The Context of Radicalization and the Variable Self: Exploring Radicalization as an Empirical Topic*.
