# Supplementary material for: The Context-Variable Self and Autonomy: Exploring Surveillance Experience, (Mis)recognition, and Action at Airport Security Checkpoints
Source: Front Psychol. 2019 Oct 22;10:2258. doi: 10.3389/fpsyg.2019.02258 (PMC6844260; doi:10.3389/fpsyg.2019.02258)
Supplement: Supplementary file 1 [file Presentation_1.pdf]

The Context Variable Self and Autonomy: Supplementary Methods Information

Meghan E. McNamara
School of Psychology and Neuroscience, University of St Andrews
mem21@st-andrews.ac.uk

1. Introduction

This manuscript contains additional information about the methods underlying the analysis reported in McNamara and Reicher (2019). Specifically, I've used this space to provide a more detailed accounting than was possible within the paper's word-limit boundaries. Word limit boundaries in psychology journals tend favor quantitative research needs; qualitative research often requires more space to explain procedures (see also, Levitt, Motulsky, Werz, Morrow, and Ponterotto, 2017, p.5). This was particularly the case for this study, as the samples were non-homogenous and the analytical methods were designed and implemented with this specific study in mind. This means that customary shorthand techniques, like simply citing Braun and Clarke (2006) as a reference for 'thematic analysis method,' were insufficient to allow readers to ascertain the quality of this paper. Therefore, I included the most critical information about the analytical procedure in the paper's methods section (McNamara and Reicher, 2019), and shifted the specialist information (as well as greater details about the data and the sample), into this supplementary materials document.[1]

It is organized in the following manner:

2. **Further information on data collection procedure**
   2.1. Diary construction information
3. **Further information on data**
   3.1. Number of reported airport security experiences
   3.2. Number of reported surveillance accounts per participant
   3.3. Time of reported surveillance experience
   3.4. Place where reported surveillance experience occurred
4. **Further information on participants**
   4.1. Age
   4.2. Student status
   4.3. Completed education
   4.4. Occupation
   4.5. Nationality
   4.6. Place of residence
   4.7. Participant race, ethnicity, or first language
5. **Further information on analytical procedure**
   5.1. The rationale for the adaptive thematic analysis procedure
   5.2. The method for the main analysis
   5.3. An explanation of the transcription, naming conventions, and the ethnographic style

[1] The choice to delineate certain methods information into 'critical' and 'specialist' categories is a value-laden analytical choice. Therefore, I used disciplinary norms and conversation with colleagues, including the empirical paper's second author, to guide me. That is how I decided which information should go in the empirical paper's methods section, and which information should be placed in this methods paper. However, be aware that I contest the notion of 'crucial' and 'specialist' methods knowledge. I would argue this categorical construction only exists because journal publication guidelines favor quantitative studies, and qualitative writers have to either adapt or not publish. This should be interrogated with more depth; unfortunately it is outside the scope of this manuscript.

2. Supplementary Information on the Data Collection Procedure

2.1 Diary Construction

The diaries were built around one prompt and two questions:

- Please tell me what happened at the airport security checkpoint.
- How did you feel about this?
- Is there anything you think I should be aware about with regard to airport security?

3. Supplementary Information About Data

3.1 Number of reported airport security experiences

These data included reports of experiences at airport security checkpoints (N = 153), examples of air travel avoidance (N = 2), and one report of a flight transfer where an officer only checked passports and boarding passes (N = 1)

3.2 Number of reported surveillance accounts per participant

Most participants (N = 33) reported two surveillance accounts. Others reported one account (N = 12), three (N = 3), four (N = 2), five (N = 3), six (N = 3), thirteen (N = 1) and fifteen (N =1) accounts.

3.3 Time of reported security experiences

Only one participant reported surveillance experiences that occurred before September 11, 2001 (N = 1; unknown date, N = 1), after which airport security procedures changed significantly (see also, Parks, 2007). For analytical clarity, only quotes from internal checkpoint accounts that occurred after 9/11 were included within this paper's analysis.

3.4 Place where reported security experiences occurred

Most (65%) of security experiences occurred in the U.S. (N = 58) and U.K. (N = 44). Twenty-seven occurred elsewhere in Europe (Germany, N = 9; Latvia, N = 5; Spain, N = 3; the Netherlands, N = 3; Lithuania, N = 2; Bulgaria, N = 1; France, N = 1; Greece, N = 1; Belgium, N = 1; Ireland, N = 1). Other screening occurred in Argentina (N = 7), Canada (N = 7), Chile (N = 2), China (N = 2), New Zealand (N = 2), Turkey (N = 1), and Morocco (N = 1). Participants also provided two general accounts attributed to no specific location (e.g., it's like this every time). One participant reported an experience in Germany at a checkpoint staffed by screeners who he said were American.

4. Supplementary Information About Participants

The study design used open categories for descriptive demographic questions (gender, age, student status, completed education, occupation, nationality, place of residence, race/ethnicity), as I collected international data. In order to balance respect for participant privacy and need to contextualize data for readers, the participants' responses to single demographic questionnaire items are summarized.

4.1 Age

Sixty-six per cent of participants were under age 29 (N = 38). Twenty-four per cent were 30-59 (N = 16), and 7% were aged 60-66 (N = 4). Two participants did not provide their age.[2] As data collection took place over a period of eight years, and I followed up with some participants (N = 14) to clarify

[2] One of them was retired, and in 2010 reported remembering when Billy Connolly started performing (1960s; Busby, 2018).

ambiguity (and, if they chose to, share new surveillance accounts, which occurred in three cases), I reported the age recorded during their first participation within the study.

4.2 Student status

Fifty-nine per cent of this study's sample (N = 34) reported that they were students. Thirty-one were enrolled in a university degree program (Bachelors, N = 16; Masters, N = 10; PhD, N = 6). A mature student, who held a previous bachelors degree, reported retraining toward an associate's degree. One participant reported independent study. Twenty-three participants were not students, and one participant did not report their student status. Most of the students reported studying Psychology (N = 9), Tourism Management and Hospitality Business (N = 7), Medicine (N = 3), Sociology (N = 2), and Economics (N = 2). Others studied Art History, Chinese Language, English, Healthcare (not specified), Linguistics, Marine Mammal Science, Photonics & Optoelectronic Devices, and Physical Drama (all N = 1). One participant was on a multiple-subject degree program (English, Film, & Psychology).

4.3 Completed education

All participants had completed secondary school. Sixty-nine per cent of participants (N = 40) had completed post-secondary education, and 26% (N = 15) had completed graduate degrees. In other words, just over two thirds of the sample previously completed at least one higher education qualification (PhD, N = 5; M.D., N = 3; Master's, N = 8; Bachelor's, N = 21; Hospital-Based Diploma in Nursing, N = 1; American Associate's Degree, N = 2; British Higher National Diploma, N = 1; British Higher National Certificate, N = 1). Eighteen participants had not completed post-secondary degrees. Sixteen of those participants were currently enrolled in university. The others were retired or had attended some university before moving on to other opportunities (both N = 1).

4.4 Occupation

Forty-five per cent of the sample (N = 26) reported employment (employed and not a student, N = 19; employed student, N = 7). One participant was an unemployed recent graduate, and two were retired. Two participants did not report this information. Employed participants held a wide range of jobs. Six participants reported employment at a post-secondary education institution (Research Fellow, N = 2; Academic – not specified, N = 1; Assistant Registrar, N = 1; Lecturer – Psychology, N = 1; Graduate Teaching Assistant, N = 1). Five participants worked in health and social care (Physician – not specified, N = 2; Medical Oncologist, N = 1; Occupational Therapy Assistant, N = 1; Social Care Worker, N = 1). Three participants worked in management (Director of National Sales, N = 1; Financial Director, N = 1; Regional Manager of a pharmaceutical company, N = 1), and two worked in other business roles (Business Owner, N = 1; Investor, N =1). Two participants were administrators in an unspecified industry, and two participants were housewives. Other participants reported employment as a Buddhist monk, music producer, and nail technician/freelance actor (all N = 1). Three participants reported "work" without specifying their occupation, and two participants did not provide this information.

4.5 Nationality

Participants reported numerous nationalities: American (N = 15), German (N = 10), British (9), Latvian (N = 6), Russian (N = 3), and Canadian (N = 2). The others were from Chile, China, Greece, Ireland, India, Italy, New Zealand, the Netherlands, Taiwan and Ukraine (all N = 1). Three reported dual nationality (Ireland & U.S., N = 2; Greece & U.K., N = 1).

4.6 Place of residence
Most participants lived in Scotland (N = 20), the United States (N = 16), Latvia (N = 8), and England (N = 8). Others lived in Germany (N = 3), Austria (N = 1), and Taiwan (N = 1). One participant simply reported their residence as 'U.K.'

4.7 Participant race, ethnicity, or first language
Most participants self-identified as White or Caucasian (N = 29) without any modifying terms. Eight Latvian residents reported Russian first language. Others reported Asian, Asian Indian (North India), British, Caucasian & American Indian, Caucasian / Irish American, Chinese, German & Cuban, Latin American, Scottish, Scottish & Indian, Taiwanese, Vietnamese, White – British, White – German, White – Irish, White – Latvian, White – Non-Hispanic, and White – Scottish (All N = 1). Three participants did not respond to this demographic question.

5. Supplementary Information on Analytical Method

5.1 Adaptive Thematic Analysis Procedure
The adaptive thematic analysis (see Table 1 in McNamara & Reicher, 2019) consisted of a framework of steps I set out in advance, which were then adapted as necessary; all adaptations were recorded and justified. I chose an adaptive approach because following thematic analysis (e.g., Braun & Clark, 2006) as a step-by-step procedure ('cookbook analysis;' Maxwell, 2013) did not facilitate the necessary depth for answering the research questions.

This study's adaptive approach used elements found in resources like Braun & Clark (2006), Boyatzis (1998), Charmaz (2006), Elliot, Fischer, & Rennie (1999), and Maxwell (2013). It also included steps that balanced pre-determined systematic procedure with flexible innovation as needed. The latter was particularly helpful because the interviews and diaries used open questions that did not presuppose what form answers might take.

Therefore, the preliminary analysis also included an emic coding step: travel security experiences were defined by 'type' in the participants' own words (e.g., okay, nervous, a nightmare). Those emic codes were then aggregated into groups of similar experiences, in order to better understand contextual differences (e.g., "I was anxious, as usual, when getting through security" was placed with "traveling now is so much more stressful [than before 9/11]").

In addition, connection mapping developed during the coding process as a sense-making tool, in order to visualize and test category relationships against divergent data. This occurred in order to examine category fit, and involved creating 'analytical trees' that linked related concepts.[3] This clarified the various contexts by illuminating similarities and differences between 'type,' which eased the subsequent analysis. The final steps involved interrogating the analytical trees in terms of the resulting categories, theme production, analysis auditing (Elliot, Fischer, & Rennie, 1999), applying the themes to the research question after a thematic literature review, and then theme refinement based on this etic understanding. This analytical process supported flexible project development (essential to the exploratory question) within a systematic and logical framework (essential to good research practice).

[3] Braun and Clark (2006) included a similar process within their method, but they placed it within their 'searching for themes' stage. The present study used connection mapping during the initial coding process. The distinction is shared for the sake of methodological clarity.

5.2 Main Analysis Procedure
The thematic analysis findings provided the foundation for the main analysis.

- The thematic analysis demonstrated a number of experience elements, including inconvenience (e.g., waiting, unpacking), worry (e.g., uncertainty what to do, concern about missing a flight), gaze (e.g., who is looking at me, what do they see), legitimacy (e.g., does this work), and recognition (participant self-definitions are affirmed) (McNamara, 2010; McNamara, 2012).

The main analysis focused specifically on recognition/misrecognition. As with the thematic analysis, I constructed a procedure, with the understanding that data immersion may well change contextual understanding, and require adaptation (see Table 2 in McNamara & Reicher, 2019). To start, I conducted theory-based coding to identify recognition and misrecognition experiences, with specific attention to participants' own analytical categories (e.g., emic distinctions; Harris, 1968):

- Recognition: Participant self-definitions and surveillance-imposed self-definitions are consonant
- Misrecognition: Participant self-definitions and surveillance-imposed self-definitions are dissonant

In order to answer the research questions, it was necessary to understand how the self-definition categories functioned within the screening experiences, the context(s) participants drew on to anchor those categories, and the experiential and action consequences associated with self-definition recognition/misrecognition. Therefore, the main analysis had three major components:

- Exploring the checkpoint as a context
- Exploring participant context
- Exploring how participants experienced recognition/misrecognition during screening

To understand the checkpoint context, I explored airport security as a social practice. This included examination of its system (e.g., procedures), structure (e.g., the technology), and how it was situated in the broader contemporary world (e.g., current events). The analysis also required understanding the self-definitions and contexts participants experienced during airport screening. Specifically, this involved coding the recognition/misrecognition extracts for self-definition categories, how recognition/misrecognition was experienced, and actions.

These extracts were then situated within each participant's other data to explore their context(s). For instance, this included referring back to other text (e.g., the whole interview or the participant's other diaries) or conducting follow-up interviews. Finally, I developed an analytical structure that answered the research question, and then referred back to the data once more to search for divergent cases that might not match this theory. The analytical structure was confirmed after ensuring there were no divergent cases, and then having a validation conversation with the paper's second author. Then, the analysis was written.

5.3 On transcription, names, and ethnographic style
Three of the methods choices used in this paper (participant quotes, pseudonyms, and the paper's ethnographic style) are worth explaining in more detail, as they are unconventional in terms of contemporary psychology research report norms.

- *Quote transcription and integration:* Since this set of airport security screening accounts included written and spoken accounts from an international sample, I paid careful attention to how to integrate these data into this ethnography. Quotes were reported as they were communicated in the diaries and interviews. Quote reporting (APA, 2009) and clarification (MLA, 2016) conventions were used where appropriate. My engagement with two different style guides was an analytical choice, and therefore should be explained.

  While the APA (2009) and MLA (2016) guides are broadly similar in terms of how their advice about integrating quotations, the way the APA guide framed their text clarification guidance is inconsistent with good practice for qualitative analyses. Specifically, the APA (2009) publication manual explains the usage of *'sic'* for text that may confuse readers as follows: "if any *incorrect* [emphasis added] spelling, punctuation, or grammar in the source might confuse readers, insert the word *sic* (p.172)." However, it is analytically problematic to categorize participant communication as incorrect in a study about how people experience their self-definitions.

  Therefore, I used the MLA (2016) guide for text clarification. MLA (2016) explains the usage of *'sic'* as something to "assure readers that the quotation is accurate even though the spelling or logic might make them think otherwise (p.86)." This construction focuses the textual intervention's logic around academic convention instead of categorizing participant communication as 'incorrect.' Therefore, I used *'sic'* to annotate unconventional spelling and capitalization in quotes taken from written material. This kept the boundary between participant expression and potential researcher error clear. However, participant syntax and word choice were reported without any editorial intervention when they were pulled from transcribed interviews, because there is good evidence to show dialect (as one but example), may be identity-relevant: vernacular can convey meaning (e.g., Hurston, 2018).[4]

- *Pseudonyms:* I used pseudonyms in the write-up rather than designations like participant one. Here, the pseudo-objective distance offered by de-naming and numbering participants would serve no analytical or narrative purpose. As names convey meaning, I asked participants to provide a pseudonym. For those that did not choose a name, I allocated them one based on naming conventions common to their region of origin and birth year. I found this information in online government databases or with support from local research assistants.

- *Ethnographic style:* I used an ethnographic style in order to ground and situate concepts, data, analysis, and theory, which were then integrated into a narrative format. The conventions of storytelling can support research communication when used with analytical intention (cf. Stoller, 1994). In that regard, I used contractions where it supported sentence rhythm and analytical clarity. This allowed better integration of active and passive voice within the manuscript, a stylistic choice that is consonant with ethnography (Stoller, 1994) and Billig's (2013) advice to communicate research in ordinary language when possible and appropriate (e.g., 'everyday English,' Orwell, 1946/1968).

[4] This is not to say that dialect conveyed meaning that was relevant to the recognition/misrecognition analysis, but simply to make the point that (particularly in a paper that deals with identity) it is philosophically unsound to imply that participant communication is 'incorrect.' That is the implication of using *'sic'* in quotes taken from spoken interview transcripts.

6. Conclusion

To sum, this document provided more detailed methods information than was possible to deliver in McNamara and Reicher (2019). Specifically, I provided additional description of the data and the sample, as well as further particulars on the adaptive thematic analysis and ethnography procedures. This document was necessary due to word count limitations imposed by disciplinary norms that favor quantitative research reports.

7. References

APA (2009). Publication Manual of the American Psychological Association, 6th Edn, Washington DC: American Psychological Association.

Billig, M. (2013). Learn to Write Badly: How to Succeed in the Social Sciences, Cambridge: Cambridge University Press.

Boyatzis, R. E. (1998). Thematic Analysis and Code Development: Transforming Qualitative Enquiry, SAGE: Thousand Oaks.

Braun, V., and Clarke, V. (2006). Using thematic analysis in psychology. Qual. Res. Psychol. 3, 77–101. doi: 10.1191/1478088706qp063oa

Busby, M. (2018). Billy Connolly Announces Retirement From Live Performance. The Guardian. Available at: https://www.theguardian.com/culture/2018/dec/03/billy-connolly-announces-retirement-from-live-performance (Accessed on April 15, 2019)

Elliot, R., Fischer, C. T., and Rennie, D. L. (1999). Evolving guidelines for publication of qualitative research studies in psychology and related fields. Br. J. Clin. Psychol. 38, 215–229. doi: 10.1348/014466599162782

Harris, M. (1968). The Rise of Anthropological Theory: a History of Theories of Culture. New York, NY: Thomas Y Crowell Company.

Hurston, Z. N. (2018). Barracoon: The Story of the Last "Black Cargo", New York, NY: Amistad/Harper Collins.

Levitt, H. M., Motulsky, S. L., Wertz, F. J., Morrow, S. L., and Ponterotto, J. G. (2017). Recommendations for designing and reviewing qualitative research in psychology: promoting methodological integrity. Qual. Psychol. 4, 2–22. doi: 10.1037/qup0000082

Maxwell, J. A. (2013). Qualitative Research design: An Interactive Approach, 3rd Edn, Los Angeles, CA: SAGE.

McNamara, M. E. (2010). An Analysis of Travel Security Experiences: What Happens at Airports? Sociology Master's Dissertation, University of Cambridge.

McNamara, M. E. (2012). Exploratory Investigation of Travellers' Experiences with Airport Security. Psychology Master's Dissertation, University of St Andrews, Scotland.

McNamara, M. E. and Reicher, S. D. (2019). The context variable self and autonomy: exploring surveillance experience, (mis)recognition, and action at airport security checkpoints. Front Psychol, 10: 2258 doi: 10.3389/fpsyg.2019.02258

MLA (2016). The MLA Handbook, 8th Edn, New York, NY: The Modern Language Association of America.

Orwell, G. (1946/1968). "Politics in the English language," in The Collected Essays, Journalism and Letters of George Orwell Volume IV: In Front of Your Nose, 1945-1950 127-139), eds S. Orwell & I. Angus (London: Secker & Warburg).

Stoller, P. (1994). Ethnographies as texts/ethnographers as griots. Am. Ethnol. 21, 353–366. doi: 10.1177/1363459308094422
